# Supplementary material for: Olympic combat sports and mental health in children and adolescents with disability: A protocol paper for systematic review
Source: PLoS One. 2025 Feb 10;20(2):e0301949. doi: 10.1371/journal.pone.0301949 (PMC11809909; doi:10.1371/journal.pone.0301949)
Supplement: S1 Checklist — (DOCX) [file pone.0301949.s001.docx]

# Reporting checklist for protocol of a systematic review and meta analysis.

Based on the PRISMA-P guidelines.

## Instructions to authors

Complete this checklist by entering the page numbers from your manuscript where readers will find each of the items listed below.

Your article may not currently address all the items on the checklist. Please modify your text to include the missing information. If you are certain that an item does not apply, please write "n/a" and provide a short explanation.

Upload your completed checklist as an extra file when you submit to a journal.

In your methods section, say that you used the PRISMA-Preporting guidelines, and cite them as:

Moher D, Shamseer L, Clarke M, Ghersi D, Liberati A, Petticrew M, Shekelle P, Stewart LA. Preferred Reporting Items for Systematic Review and Meta-Analysis Protocols (PRISMA-P) 2015 statement. Syst Rev. 2015;4(1):1.

|  |  | Reporting Item | Page Number |
| --- | --- | --- | --- |
| **Title** |  |  |  |
| Identification | [#1a](https://www.goodreports.org/reporting-checklists/prisma-p/info/#1a) | The study has been identified as a protocol for a systematic review. | 1 |
| Update | [#1b](https://www.goodreports.org/reporting-checklists/prisma-p/info/#1b) | This is not an update of a previous review, so this item is not applicable. | n/a |
| **Registration** |  |  |  |
|  | [#2](https://www.goodreports.org/reporting-checklists/prisma-p/info/#2) | The review is registered with PROSPERO, registration number CRD42023452489. | 5 |
| **Authors** |  |  |  |
| Contact | [#3a](https://www.goodreports.org/reporting-checklists/prisma-p/info/#3a) | Authors, affiliations, and contact information are provided. | 1 |
| Contribution | [#3b](https://www.goodreports.org/reporting-checklists/prisma-p/info/#3b) | Contributions of protocol authors are outlined; the guarantors of the review are Dr. Janet Hauck and Dr. Simone Ciaccioni, given the correspondence address. | 1, 22 |
| **Amendments** |  |  |  |
|  | [#4](https://www.goodreports.org/reporting-checklists/prisma-p/info/#4) | If there are amendments to this protocol, plan to document important protocol amendments in future updates or correspondences. | n/a |
| **Support** |  |  |  |
| Sources | [#5a](https://www.goodreports.org/reporting-checklists/prisma-p/info/#5a) | No specific funding received for this study. | 1 |
| Sponsor | [#5b](https://www.goodreports.org/reporting-checklists/prisma-p/info/#5b) | Not applicable. | n/a |
| Role of sponsor or funder | [#5c](https://www.goodreports.org/reporting-checklists/prisma-p/info/#5c) | Not applicable. | n/a |
| **Introduction** |  |  |  |
| Rationale | [#6](https://www.goodreports.org/reporting-checklists/prisma-p/info/#6) | The rationale for the review, including the importance of mental health among children and adolescents with disabilities and the potential impact of Olympic combat sports, is well-described. | 4-5 |
| Objectives | [#7](https://www.goodreports.org/reporting-checklists/prisma-p/info/#7) | The objective is clearly stated, focusing on how participation in Olympic combat sports influences mental health outcomes in this population. | 5 |
| **Methods** |  |  |  |
| Eligibility criteria | [#8](https://www.goodreports.org/reporting-checklists/prisma-p/info/#8) | Study characteristics, including PICO (participants, interventions, comparators, outcomes), study design, setting, time frame, and report characteristics, are specified. | 6-7 |
| Information sources | [#9](https://www.goodreports.org/reporting-checklists/prisma-p/info/#9) | Intended information sources such as electronic databases are described. | 11 |
| Search strategy | [#10](https://www.goodreports.org/reporting-checklists/prisma-p/info/#10) | A draft search strategy for at least one database, including planned limits to ensure reproducibility, is provided. | 11 |
| Study records - data management | [#11a](https://www.goodreports.org/reporting-checklists/prisma-p/info/#11a) | EndNote x9 and Covidence are mentioned for managing records and data. | 13 |
| Study records - selection process | [#11b](https://www.goodreports.org/reporting-checklists/prisma-p/info/#11b) | A two-phase screening process involving independent reviewers is outlined. | 13 |
| Study records - data collection process | [#11c](https://www.goodreports.org/reporting-checklists/prisma-p/info/#11c) | The method for data extraction, including independent extraction and consensus, is described. | 15-16 |
| Data items | [#12](https://www.goodreports.org/reporting-checklists/prisma-p/info/#12) | Variables for which data will be sought are listed, including PICO items and comparators. | 6-7 |
| Outcomes and prioritization | [#13](https://www.goodreports.org/reporting-checklists/prisma-p/info/#13) | Outcomes for data collection are listed. | 7-10 |
| Risk of bias in individual studies | [#14](https://www.goodreports.org/reporting-checklists/prisma-p/info/#14) | Methods for assessing the risk of bias, including tools like Rob 2.0 and ROBINS-I, are described. | 16-17 |
| Data synthesis | [#15a](https://www.goodreports.org/reporting-checklists/prisma-p/info/#15a) | Our protocol mentions the intention to use meta-analytic techniques if the data from the included studies exhibit sufficient homogeneity. This indicates a criterion for quantitative synthesis—homogeneity among studies. | 17 |
| Data synthesis | [#15b](https://www.goodreports.org/reporting-checklists/prisma-p/info/#15b) | For quantitative synthesis, we plan to employ both frequentist and Bayesian approaches, using JASP software. The frequentist approach involves calculating the standardized mean difference (SMD) and its 95% confidence intervals (CIs), controlling for baseline differences. This outlines the planned summary measures (SMD and CIs) and methods of handling and combining data from studies. Heterogeneity will be assessed using Cochrane's Q and I2 statistics. | 18 |
| Data synthesis | [#15c](https://www.goodreports.org/reporting-checklists/prisma-p/info/#15c) | Additional analyses such as sensitivity or subgroup analyses, and moderation analysis is proposed to explore the impact of various factors like age, type or level of disability, and intervention characteristics on the outcomes. | 16, 19-20 |
| Data synthesis | [#15d](https://www.goodreports.org/reporting-checklists/prisma-p/info/#15d) | If quantitative synthesis is not appropriate due to high heterogeneity or insufficient data, we plan to synthesize findings narratively. This is briefly mentioned with the use of the Synthesis without Meta-Analysis (SWiM) tool for data that cannot be included in the meta-analysis. | 2 |
| Meta-bias(es) | [#16](https://www.goodreports.org/reporting-checklists/prisma-p/info/#16) | Plans for assessing meta-bias, such as publication bias, are specified. | 18 |
| Confidence in cumulative evidence | [#17](https://www.goodreports.org/reporting-checklists/prisma-p/info/#17) | We will use the GRADE methodology to assess the quality of the evidence for each key outcome. | 18-19 |

The PRISMA-P elaboration and explanation paper is distributed under the terms of the Creative Commons Attribution License CC-BY. This checklist was completed on 30. November 2022 using <https://www.goodreports.org/>, a tool made by the [EQUATOR Network](https://www.equator-network.org) in collaboration with [Penelope.ai](https://www.penelope.ai)
