# Supplementary material for: Olympic combat sports and mental health in children and adolescents with disability: A protocol paper for systematic review
Source: PLoS One. 2025 Feb 10;20(2):e0301949. doi: 10.1371/journal.pone.0301949 (PMC11809909; doi:10.1371/journal.pone.0301949)
Supplement: Attachment — Submitted filename: Response to Reviewers.docx [file pone.0301949.s003.docx]

**Response to Reviewer 1’s comments:**

Thank you sincerely for your valuable time and constructive comments and suggestions. We have considered all the comments carefully and revised our manuscript accordingly. Our responses to each comment (shown in Italics) are as follows.

|  | **Comment** | **Response** |
| --- | --- | --- |
| **1** | *The objective of the study is, through a systematic review protocol, to outline the rationale and methodology for investigating how participation in Olympic combat sports influences the mental health outcomes of this population. The study is characterized as Reviewing Study Protocols.* | We understand the concern of the reviewer and thank for sharing it with us. After acceptance of the PROSPERO registration, and while writing the protocol paper, we have already run the entire search and performed two steps of preliminary analyses, which have been presented at two international conferences in the US (exercise and sport psychology; May 2024) and in Europe (sports sciences, July 2024), respectively.  We can anticipate that our review retrieved 12 studies (six RCTs and CTs, respectively) delving into the role of Olympic combat sports as a rehabilitative tool. The above 12 intervention studies involved 436 mostly novice and male children/early adolescents (age: 11.4±2.8 years) practicing karate (n=5), judo (n=3), karate and judo (n=2), boxing (n=1), and taekwondo (n=1). Cognitive (i.e., autism spectrum and learning disorders) and physical (e.g., developmental coordination disorders) disabilities were represented.  Thus, while the number of pertinent studies is not high, we deemed it sufficient to draw first conclusions. Our analysis indicates that Olympic combat sports, notably karate and judo, are beneficial in enhancing social-emotional functioning in children with autism. Furthermore, karate and judo positively impacted aggression and self-esteem in children with learning disabilities.  Furthermore, we believe that these preliminary outcomes can confirm the usability of the protocol. The synthesis of the studies selected according to this protocol can provide an outlook to inform the development of future research targeted to fill existing gaps in this still underrepresented but socially relevant area of research. |
| **2** | *Although I recognize that the study plays an excellent role considering methodological rigor and quality from the search strategy, screening process, data extraction, data aggregation, analysis of risk of bias and methodologic quality, and data analysis (including possible meta-analysis), it was not applied in a real situation. This aspect is important, especially because the chosen topic is very specific - Olympic combat sports and mental health in children and adolescents with disability. There is a range of studies on combat sports, but when we stratify into children and adolescents this number decreases a lot and then when we stratify into disabilities and then mental health, even more so. The fact is that I'm not sure if the excellent proposed protocol is useful and can be used in practice due to the very low number of studies on this specific subject. Therefore, I strongly recommend that authors use the protocol and test its usability. Only then will we be able to see the importance of study for science.* |  |

**Response to Reviewer 2’s comments:**

Thank you sincerely for your valuable time and constructive comments and suggestions. We have considered all the comments carefully and revised our manuscript accordingly. Our responses to each comment (shown in Italics) are as follows.

|  | **Comment** | **Response** |
| --- | --- | --- |
| **1** | *The manuscript is not original study and not exactly review, it is proposition for kind of procedure. The topic is important (mental health). The weakness is the selection of literature. There are a lot of self-citations.* | Thank you very much for your valuable feedback on our manuscript. And we agree that the publication of protocol papers is still an issue of debate with pros and cons of the separate or joint publication of the protocol and the review results.  We reflected on this issue and decided to adhere to the view that publishing a protocol paper enhances transparency and reproducibility. While transparency is already ensured by the PROSPERO registration, the publication of a protocol paper trades the parsimoniousness of a joint paper for the possibility to provide more details for the reproducibility of this systematic review. We believe that the reproducibility is especially relevant when the area of research is relatively under-investigated and the review conclusions will likely call for future research and evidence synthesis.  We can anticipate that this is the case of our systematic review. After acceptance of the PROSPERO registration, and while writing the protocol paper, we have already run the entire search and performed two steps of preliminary analyses (presented in 2024 at two international conferences in the US and Europe). Our review retrieved 12 studies (six RCTs and CTs, respectively) delving into the role of Olympic combat sports as a rehabilitative tool. Analyses allowed us to draw some conclusions, but also highlighted gaps in need for future research.  In addition, we are grateful for this indication which allows us better balancing citations and related references, as it is not our habit to exceed in self-citations. We have checked all citations/references and have deleted 3 of 6 self-citations. Specifically, we have deleted those self-citations/references regarding judo studies with older participants (page 3, lines 59, 61; page 4, line 68) that had been cited in support of statements for the general population. |

**Response to Reviewer 3’s comments:**

Thank you sincerely for your valuable time and constructive comments and suggestions. We have considered all the comments carefully and revised our manuscript accordingly. Our responses to each comment (shown in Italics) are as follows.

|  | **Comment** | **Response** |
| --- | --- | --- |
| **1** | *The authors demonstrated an interesting protocol for the systematic literature review regarding Olympic combat sports and mental health parameters among children and adolescents with disabilities. In addition, considering the robust, thorough, and high-quality methodological aspects, the future study will have the potential to be published in some of the most prestigious scientific journals in the area of sports sciences and applied exercise psychology. For these reasons, a minor revision of this study protocol is recommended! However, the stated comments and objections should improve the current version of the manuscript.* | We are grateful to the Reviewer for their positive judgment of the methodological rigor of our protocol. Indeed, we did our best to ensure the best possible conditions to perform a robust and above all replicable systematic review. |
| **2** | *The suggested study title: Olympic Combat Sports and Mental Health in Children and Adolescents with Disabilities: A Protocol for Systematic Review.* | Thank you for this suggestion, which ameliorates the informative value of the title and its identifiability as a systematic review in database searches. We have integrated the title accordingly (Page 1). |
| **3** | *Regarding the rationale of this investigation, it is recommended to emphasize potential innovative aspects of the future systematic review referring to Olympic combat sports and mental health. Expressly, will the literature synthesis approach only confirm the obtained results of the existing studies, or several new findings will be provided? Please report it, if possible.* | Thank you for this request aimed at highlighting the novelty of this review. We have tried to strengthen the rationale by emphasizing that the literature review is not only targeted to synthesize the existing evidence. It is also and above all targeted to identify which consistencies in results may allow to draw first conclusions with applied implications, and which gaps call for future research on the nuances of the OCS-mental health relation in children and adolescents with disabilities (page 5, lines 109-113). |
| **4** | *The name of the subheading "types of studies" is entirely inappropriate. The authors should highlight that this subheading is related to the "study design"! In addition, do controlled trials (CTs) pertain to non-randomized controlled investigations? It is highly desirable to employ suggested terminology across the whole manuscript. Lastly, the text concerning the last claim in this subheading is redundant and should be presented within the first statement.* | The subheading has been renamed as requested (page 7, line 153).  Also, as properly requested, we use the terms randomized controlled trials (RCTs) and non-randomized controlled trials (non-RCTs) consistently throughout the manuscript and have deleted the term ‘controlled trials’ that we had unclearly used to indicate non-randomized trials.The full terms, followed by the abbreviations within brackets, are introduced the first time these terms occur in the manuscript (page 2, lines 31-32). Thereafter, we use only the acronyms (RCTs and non-RCTs; page 7, line 157; page 17, line 367; page 18, line 382).  We agree that the text concerning the last claim in this subheading is redundant: it should be deleted from there and merged with the first statement. Thank you for the attentive check: we have done it (page 7, lines 154-156). |
| **5** | *Please indicate whether the comparison groups will include children and adolescents with disabilities or their typically developing counterparts. Please highlight the missing facts!* | We apologize for having chosen the wrong place to include the information on whether the comparison groups will include children and adolescents with disabilities or their typically developing counterparts. We had indicated it in the ‘Data synthesis’ section (2.11: “...encompassing populations both with and without disabilities.” (page 18).  We have now expanded this information and included it in the ‘Comparators’ section (2.4) as follows (page 10, lines 228-237) “Three primary comparison groups including age groups of children and/or adolescents with or without disabilities will be considered:...”.  More generally, we agree that the difference among comparison group types is an issue that deserves more attention.  In addition to the overall distinction of comparator groups that holds for both interventional and non-interventional studies (page 11, lines 239-244), we have now made a further distinction specific for intervention studies (page 11, lines 244-247). Indeed, an overall issue is the Hawthorne effect, which has been shown to lead to larger effects in meta-analyses when the comparator is a passive group, compared to active controls involved in any specific activity alternative to the intervention (e.g., Ciria et al., 2023). Beyond this overall issue, specifically as concerns physical activity/sport interventions, there is increasing attention to the different expectancy value for improvement of different physically and non-physically active control activities which may lead to different results. For example, as regards cognitive outcomes (Ziv et al., 2024), non-physically active video-lecture seem to generate similar expectation to improve cognition as physically activities as walking or stretching, whereas activities as yoga or meditation may generate a higher expectancy for improvement.  Thus, we have now added a shorter version of this information (and Ziv et al.’s reference) to indicate that we will include any type of comparison group, categorizing them as passive, non-physically active and physically active and considering the eventual differences that may arise from using different comparison groups.   - Ciria, L. F., Román-Caballero, R. R., Vadillo, M. A.,. Holgado, D., Luque-Casado, A., Perakakis, P., & Sanabria, D. (2023). An umbrella review of randomized control trials on the effects of physical exercise on cognition. Nature Human Behaviour, 7, 928-941. - Ziv, G., Levin, O., & Netz, Y. (2024). Selecting an appropriate control group for studying the effects of exercise on cognitive performance. Psychology of Sport and Exercise, 72, 102602. |
| **6** | *How do you intend to extract data from the figures across the included studies? Please specify.* | As regards the way to extract data from figures, we have added that authors will be contacted and kindly asked to provide the data for review purposes (page 15, lines 322-326). |
| **7** | *The text concerning certainty of evidence (GRADE assessment) must be presented as a separate subheading. Please provide these corrections!* | Thank you for providing such detailed feedback regarding the presentation of the certainty of evidence (GRADE assessment). We have made the necessary corrections and have now presented the GRADE assessment under a separate subheading as requested. (page 19, line 410-420). |
| **8** | *Based on the relevant findings, will the future systematic review have significant practical implications? Please emphasize and discuss!* | Thank you for asking us to underline the potential practical implications of our future review paper. We have added them at the end of the discussion section (page 22, lines 495-502). |
| **9** | *The manuscript contained several confusing statements and unsuitable expressions (lines 80-82; line 221). Please make the text fluent and improve your terminology!* | Thank you for your valuable feedback.We have carefully reviewed the highlighted sections, and made the necessary corrections to enhance the clarity, fluency, and appropriateness of the terminology used. We appreciate your guidance in improving the quality of our work and have ensured that the revised text addresses the concerns raised (page 4, lines 82-86; page 11, lines 255-257). |
